# The Accuracy of Jaws Repositioning in Bimaxillary Orthognathic Surgery in Patients with Cleft Lip and Palate Compared to Non-Syndromic Skeletal Class III Patients

**DOI:** 10.3390/jcm11092675

**Published:** 2022-05-09

**Authors:** Benedetta Bollato, Martina Barone, Antonio Gracco, Ugo Baciliero, Giorgia Crivellin, Giovanni Bruno, Alberto De Stefani

**Affiliations:** 1 Department of Neuroscience, School of Dentistry, University of Padua, 35100 Padua, Italy; bollato.benedetta@gmail.com (B.B.); martinabarone4@gmail.com (M.B.); antoniogracco@gmail.com (A.G.); crive.giorgia@gmail.com (G.C.); giobruno93@gmail.com (G.B.); 2 Maxillofacial Surgery Complex Unit, San Bortolo Hospital of Vicenza (Italy), 36100 Vicenza, Italy; baciliero@studiobaciliero.com

**Keywords:** digital surgical planning, cleft, orthognathic surgery, skeletal class III

## Abstract

**Background**: The present study aims to compare the accuracy of jaw repositioning in bimaxillary orthognathic surgery using digital surgical planning in cleft lip and palate patients and in non-syndromic skeletal class III patients in order to investigate if orthognathic surgery achieves different results in the first group of patients. **Method**: This study included 32 class III adult patients divided into 2 groups: cleft lip and palate (A, *n* = 16) and non-cleft (B, *n* = 16). For each patient, a 2D pre-surgical visual treatment objective was performed by the surgeon to predict hard tissue changes, and the surgical outcome was compared with that planned by using cephalometric measurement (ANB, SNA, SNB, Ar-Go-Me, S-Ar-Go). The statistical analysis showed equivalence between obtained and planned results for each measurement both in group A and in group B, but the difference between the planned and the obtained result was smaller in group B regarding ANB angle. **Conclusions**: Digital surgical planning ensures better predictability of the surgical results and higher accuracy of surgery in complex patients, such as those with cleft lip and palate.

## 1. Introduction

Maxillary growth deficiency among patients with cleft lip and palate (CLP) is well documented in the literature. While the major cause of Class III malocclusion in non-CLP subjects is hereditary, multiple causes are implied in the development of skeletal Class III in CLP patients, and they can be divided into intrinsic and extrinsic [1,2,3,4,5].

Intrinsic causes are related to developmental deficiency, inheritance, deficiency of the alveolar bone due to missing teeth, and mouth breathing caused by the obstruction of the nasal passage [6,7].

Iatrogenic factors are related to surgical repair and to the subsequent scar formation and contracture in the growth centers of the maxilla. The importance of these factors is demonstrated by the normal maxilla growth in patients who were not operated on during childhood [4].

The severity of maxillary hypoplasia appears different in unilateral and bilateral cleft cases. In the former, the maxillary midline deviates toward the side of the cleft, and the smaller maxillary segment is generally dislocated superiorly, posteriorly, and medially. In bilateral CLP cases, the premaxilla appears often protruded and may be either superiorly or inferiorly positioned. Many patients have a posterior cross-bite due to the medial collapse of the alveolar bone in that site [8].

Children born with CLP receive approximately 8.6 surgical procedures before adulthood within a treatment plan that includes a thoughtfully staged reconstruction [9].

Patients in this study were treated at San Bortolo Hospital of Vicenza (Vicenza, Italy), where the treatment protocol of maxillofacial surgery adopted in the rehabilitation of CLP consists of a first phase of pre-surgical maxillary orthopedic management with the appliance of a palatal orthopedic plate in 1-week-old children, then patients undergo soft palate surgery at 3 months. Lip and nose correction is performed when children are 6 months old, and the hard palate is repaired at 18 months. Speech and language therapy assessments usually take place at 20–24 months with soft palate exercises and within 3–4 years with logopedic therapy, if necessary. Orthodontic treatment, which helps to improve the alignment and appearance of teeth, may also be required from 5 to 6 years. An Alveolar Bone Graft (ABG) procedure is performed when there is insufficient bone in the area of the gum line defect in 9–10-year-old patients.

Orthodontic-surgical treatment represents the choice in skeletal class III patients with moderate or severe malocclusion when orthodontic treatment is not sufficient to resolve the malocclusion. The incidence of the need for orthognathic surgery among CLP patients is highly variable depending on the institution and investigator, and it is reported to vary from 30% to 75% of all CLP patients [10,11,12,13,14]. 

The pre-surgical orthodontic treatment aims to align and level the dentition and remove dental compensations to prepare the dental arches for subsequent surgical repositioning [15].

Orthognathic surgery is performed when the deciduous exfoliation has ended, the arches have been leveled and aligned, and the growth of the jaws is complete [16]. 

CAD/CAM technology and 3D computer-aided design systems have led to the introduction of virtual surgical planning (VSP) in orthognathic surgery (OGS). VSP allows the surgeon to analyze patient-specific anatomical discrepancies and to evaluate different surgical approaches before entering the operating room. Several studies have shown that higher accuracy and better predictability are achieved with digital surgical planning compared to traditional approaches [17,18]. 

Patients with CLP are treated using the same surgical techniques as non-cleft patients, but orthognathic surgery appears more difficult to perform because of the scars resulting from previous surgeries, which restrict the movement of the maxilla and increase the relapse rate associated with maxillary surgical advancement; this has been reported to happen 5% to 80% of the time [19]. In CLP patients, not all surgical goals can be easily achieved due to the altered anatomy, especially in patients with a large defect. 

This study aims to demonstrate if digital surgical planning allows achieving comparable results in complex cases, such as in CLP patients, compared to non-cleft patients.

## 2. Materials and Method

This retrospective observational study was conducted on a cohort of 32 adult patients who received a bimaxillary orthognathic surgery treatment. All the surgeries were performed by the same maxillofacial surgeon at the Maxillofacial Surgery Department of the San Bortolo Hospital of Vicenza (Vicenza, Italy) from 2015 to 2021. 

Patients were divided into two different groups:

- Group A included 16 adult patients with cleft lip and palate (12 males, 4 females, mean age 20.4 years, SD 2.6);
- Group B consisted of 16 patients with a non-syndromic skeletal class III (4 males, 12 females, mean age 24.6 years, SD 5.14).

The study sample was selected following these inclusion and exclusion criteria:

- Skeletal class III (with ANB ≤ 0 and/or Wits appraisal ≤ −2 mm);
- Unilateral or bilateral cleft lip and palate (only for group A);
- Absence of congenital craniofacial anomalies or syndromes (only for group B);
- Orthodontic-surgical treatment with bimaxillary orthognathic surgery;
- Absence of previous facial traumas;
- No previous orthognathic surgery;
- Availability of adequate radiographic documentation, photographs, dental casts, pre/post cephalometric analysis, 2D pre-surgical Visual Treatment Objectives (VTO).

### 2.1. Digital Surgical Planning

The maxillofacial surgeon prepared a 2D pre-surgical VTO for each patient to predict hard tissue changes after surgery by using the Dolphin Imaging software (Dolphin Imaging & Management Solutions, Patterson Dental Supply Inc., St. Paul, MN, USA). 

**Figure 1:**
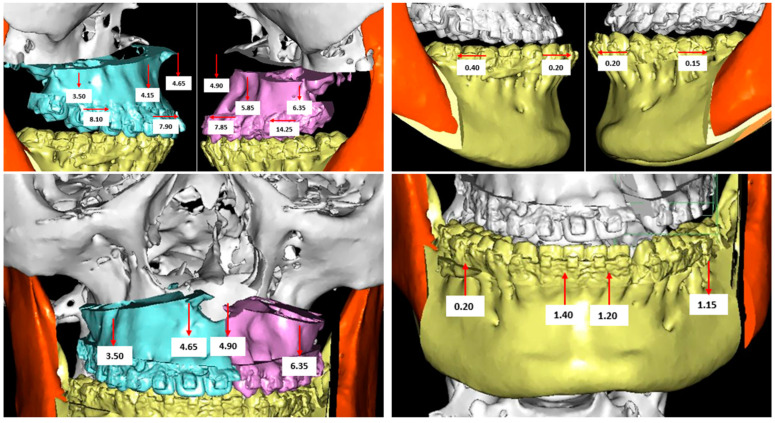
Digital surgical planning of a cleft lip and palate (CLP) patient performed with the DDS-Pro software.

**Figure 2:**
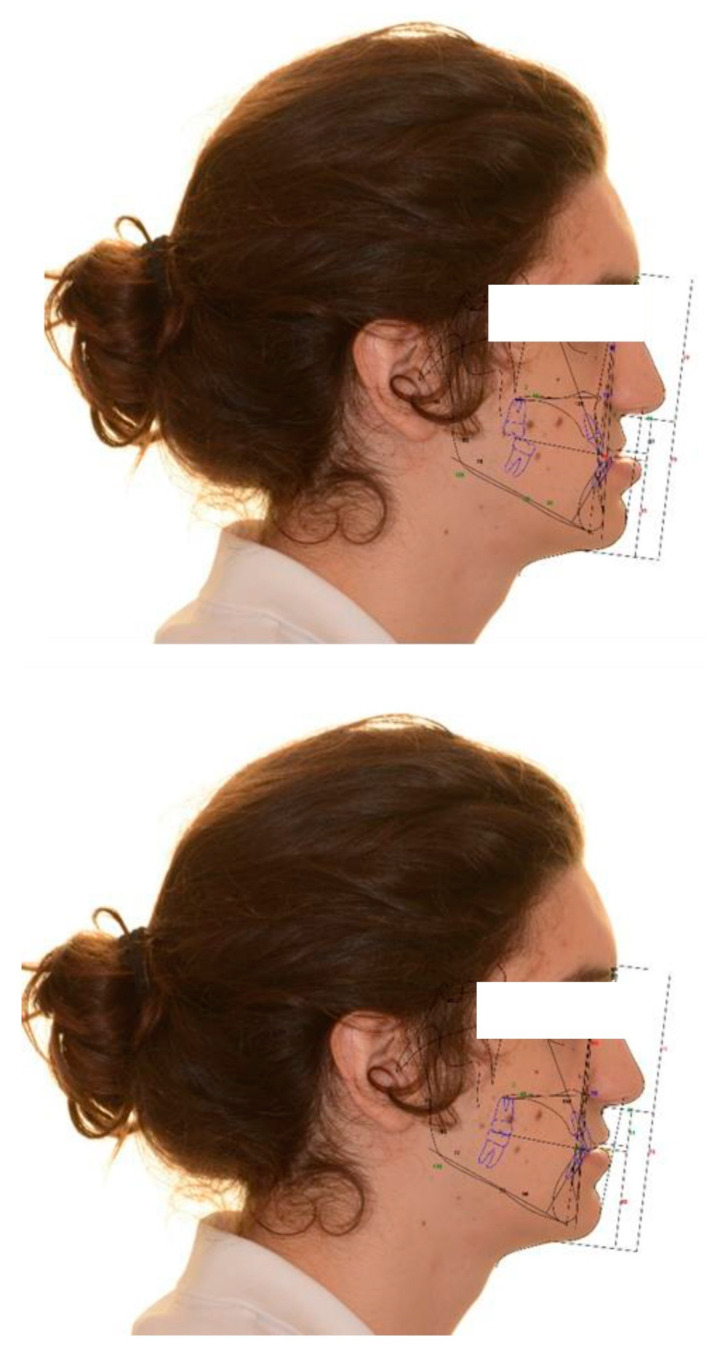
Pre-intervention cephalometry and VTO of a CLP patient. VTO: Visual Treatment Objectives; CLP: cleft lip and palate.

DDS-Pro software (DDS-Pro version 2.4.0_2020, JST sp, Częstochowa, Poland) was used for digital surgical planning (Figure 1). This software allows performing a computer-assisted surgery on the 3D virtual reconstruction of the patient’s skull. Data for the 3D reconstruction were obtained from a CBCT that all patients performed after the pre-surgical orthodontic preparation phase. To obtain information about dentition and interocclusal relationships, the patients’ dental casts were scanned to create a 3D digital model, which was integrated into the CBCT scan. The jaws osteotomies were simulated on the 3D patient’s skull reconstructions, and the bone segments were repositioned in the location established by the pre-surgical 2D VTO (Figure 2). Finally, CAD/CAM technology was used by the dental laboratory to produce the intermediate and final surgical splints. These splints were virtually designed and then manufactured with 3D printing using a rapid prototyping machine (XFAB 2500PD, DWS Systems, Thiene, Italy). The material used was DS 3000 resin, specifically created for use in the oral cavity.

### 2.2. Evaluation of the Surgical Accuracy

**Figure 3:**
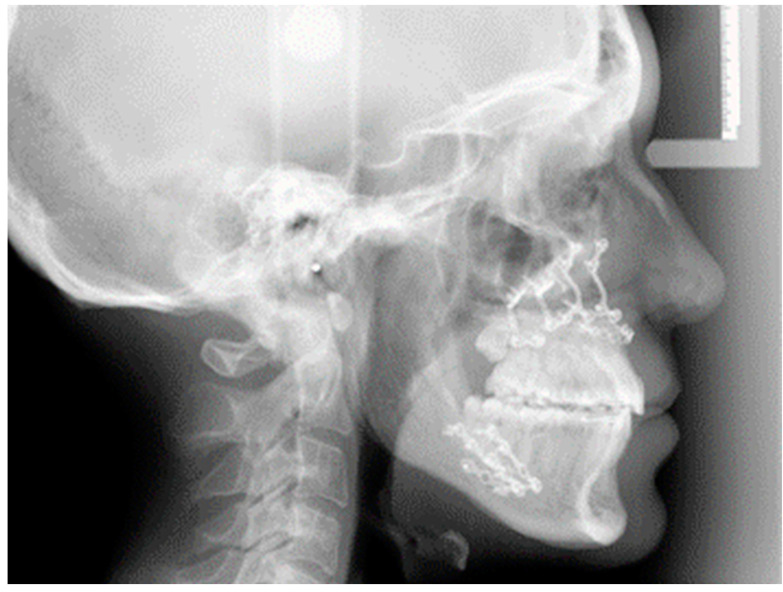
Post-surgery-lateral teleradiograph of a CLP young man.

The cephalometric values acquired by the 2D pre-surgical VTO (Figure 2) were compared with those obtained from the after-surgery cephalometry (Figure 3) to evaluate the accuracy of jaw repositioning in the two groups of patients (Roth-Jarabak analysis was used). This allowed comparing the planned surgical movements with those effectively obtained. The cephalometric measurements used were:

- ANB angle to evaluate the skeletal class;
- SNA and SNB angles for the evaluation of the anterior-posterior position of the mandible and maxilla, respectively, relative to the upper and lower jaw;
- Gonial and Articular angles to evaluate verticality.

Dolphin Imaging software (Patterson Dental Supply, St. Paul, MN, USA) was used to perform cephalometric analysis, and the cranial base was used as a reference to perform superimpositions of pre- and post-treatment.

The collected data were organized in a table (Excel, Microsoft Office 365, Microsoft, Redmond, DC, USA).

### 2.3. Statistical Analysis

To verify the equivalence of the surgical outcome between cleft and non-cleft patients, a tolerance margin of 2° was set by the authors. Sample size calculation required 16 patients in both group A and group B to demonstrate with a power of 80% and a type I error of 5% that the difference between planned and obtained results did not exceed the 73% of the standard deviation (SD). Sample size calculation was performed with the TrialSize package for R (R Foundation for Statistical Computing, Vienna, Austria). The same sample allows demonstrating, with a power of 80% and a Type I error of 5%, a standardized effect size of 1. Continuous data were expressed as mean and SD while categorical data as frequency and percentage. TOST test (two one-sided test) was used to verify the hypothesis of equivalence between planned and obtained results, and the comparison between cleft and non-cleft patient groups was performed with the two-tailed Student’s *t*-test. A *p*-value < 0.05 was considered statistically significant. R 4.1 was used to perform the statistical data analysis (R Foundation for Statistical Computing, Vienna, Austria) REF (REF: R Core Team 2021. R: A language and environment for statistical computing). 

## 3. Results

Initial characteristics of the cases included in the study, initial ANB angle, and Wits appraisal are shown in Table 1. Group A included more males and younger patients than group B, while no statistically significant differences were observed in the initial cephalometric data (Wits appraisal and ANB).

Table 2 shows the difference, in absolute value, between the obtained and planned result in cleft lip and palate patients (group A). The statistical analysis shows an equivalence (with a margin of tolerance of 2°) between obtained and planned results for all the measurements (*p* < 0.05).

Table 3 shows the difference, in absolute value, between the obtained and planned result in non-syndromic skeletal class III patients (group B). The statistical analysis shows an equivalence (with a margin of tolerance of 2°) between obtained and planned results for all the measurements (*p* < 0.05).

In general, the difference (in absolute value) between the planned and the obtained result was smaller in group B than in group A regarding ANB angle (*p* = 0.03), while it was found to be comparable between the two groups as regards the other measures. Table 4 shows the comparison between groups A and B.

## 4. Discussion

Orthognathic surgery allows reshaping the maxilla and mandible in patients with craniofacial deformities [20]. To achieve a successful outcome, it is important to adopt accurate and safe surgical techniques, adequate orthodontic treatment, and precise preoperative surgical planning [21]. Several studies have demonstrated the accuracy of 3D surgical simulations in achieving better clinical outcomes. More information can be processed using 3D surgical planning, especially in cases involving facial asymmetries such as in patients with CLP. Although asymmetry is present even in the “normal face”, it appears exaggerated in CLP patients, especially in unilateral cases. This factor makes OGS planning especially demanding [18,22,23,24,25]. 

The aim of the present study was to compare the accuracy of jaw repositioning in bimaxillary orthognathic surgery in cleft lip and palate patients and in non-syndromic skeletal class III patients to investigate if orthognathic surgery achieves different results in the two groups. The study sample appeared homogeneous in terms of initial cephalometric data (ANB angle and Wits appraisal), as these were similar in the two groups, and no statistically significant differences were observed. As regards the distribution by sex and age, group A included more males and younger patients than group B; nevertheless, this appears in accordance with the epidemiological data available in the literature. There is a male/female ratio of 2:1 as regards cleft lip associated or not with cleft palate, while isolated cleft palate occurs mostly in women [26,27].

In this study, only the hard tissues and angular cephalometric measurements were considered for the comparison because it was observed that linear measurements were more susceptible to errors. Soft tissue adaptation needs about 6–12 months from surgery to achieve complete stabilization, while the lateral radiography used for the post-operative cephalometry was performed in the immediate post-operative period.

The first part of the analysis aimed to verify the individual accuracy of the digital surgical planning by comparing it with the post-surgical result in cleft and non-syndromic skeletal class III patients. For both groups, the statistical analysis showed equivalence between the planned and the obtained result for all the cephalometric measurements considered (*p* < 0.05).

The second part of the analysis aimed to compare the accuracy of jaw repositioning in patients with cleft lip and palate and in non-syndromic skeletal class III patients. The statistical analysis demonstrated the existence of equivalence between the planned and obtained results for all the measurements considered, except for the ANB angle. In fact, this difference appeared smaller in group B if compared to group A. Although this difference appeared statistically significant (*p* = 0.03), the data have reduced clinical value because the variation between the planned and obtained ANB angle value in the two groups was only a half degree. 

This small discrepancy might be analyzed considering the complexity of orthognathic surgery in patients with CLP. Recent studies described a greater difficulty in the OGS in cleft patients with skeletal class III than in non-cleft patients because of the presence of scarring from previous surgeries, which can restrict the maxilla movement. The scarring also increases the relapse rate, which has been reported to occur 5% to 80% of the time [24,25,28].

The authors can affirm that digital surgical planning might ensure better predictability of post-surgical results and higher accuracy of surgery in complex patients, such as those with cleft lip and palate. Although orthognathic surgery appears more difficult to perform in cleft patients, the use of digital surgical planning can allow achieving comparable results in cleft and non-cleft patients. 

A possible limitation of this study is represented by the limited number of the study sample. Further studies would be desirable; a multicenter study would allow obtaining the largest sample for the study. 

## 5. Conclusions

The obtained results showed that the accuracy of jaw repositioning was comparable in the two groups. Clinically, these results show that the use of a digital approach can allow achieving comparable results in CLP patients and in III class non-syndromic patients.

## Figures and Tables

**Table 1:** Distribution of the sample based on the average age at the time of surgery, sex, and initial cephalometric values.

	*Group A: Average (SD) or n (%)*	*Group B: Average (SD) or n (%)*	*p*-Value
***n***	16	16	-
**Males**	12 (75%)	4 (25%)	0.01
***Age at the time of surgery***	20 (3)	25 (5)	0.008
**Wits appraisal, mm**	−6.9 (4.9)	−9.3 (4.8)	0.19
**ANB, °**	−4.2 (2.7)	−3.3 (4.5)	0.51

**Table 2:** Group A: cleft lip and palate patients.

	DIFFERENCE (IN ABSOLUTE VALUE) BETWEEN THE OBTAINED AND PLANNED RESULT, AVERAGE (SD)	CASES WITH ABSOLUTE DIFFERENCE > 2°, *N* (%)	EQUIVALENCE TEST: 90% CI (*p*-VALUE)
**ANB, °**	1.20 (1.01)	2 (12%)	0.76 to 1.64 (*p* = 0.003)
**SNA, °**	0.90 (0.95)	0 (0%)	0.48 to 1.31 (*p* = 0.0009)
**SNB, °**	0.66 (0.49)	0 (0%)	0.44 to 0.87 (*p* < 0.0001)
**AR-GO-ME, °**	1.09 (1.32)	2 (12%)	0.51 to 1.67 (*p* = 0.007)
**S-AR-GO, °**	0.83 (0.97)	1 (6%)	0.40 to 1.25 (*p* = 0.001)

**Table 3:** Group B: non-syndromic skeletal class III patients.

	DIFFERENCE (IN ABSOLUTE VALUE) BETWEEN THE OBTAINED AND PLANNED RESULT, AVERAGE (SD)	CASES WITH ABSOLUTE DIFFERENCE > 2°, *N* (%)	EQUIVALENCE TEST: 90% CI (*p*-VALUE)
**ANB, °**	0.54 (0.50)	0 (0%)	0.32 to 0.76 (*p* = 0.0003)
**SNA, °**	0.70 (0.59)	1 (6%)	0.44 to 0.96 (*p* = 0.0001)
**SNB, °**	0.81 (0.78)	1 (6%)	0.47 to 1.15 (*p* = 0.0004)
**Ar-Go-Me, °**	1.43 (0.98)	3 (18%)	1.00 to 1.85 (*p* = 0.02)
**S-Ar-Go, °**	0.49 (0.44)	0 (0%)	0.30 to 0.68 (*p* = 0.0002)

**Table 4:** Comparison between groups A and B.

	DIFFERENCE (IN ABSOLUTE VALUE) BETWEEN THE OBTAINED AND PLANNED RESULT, AVERAGE (SD)			-
	**Group A**	**Group B**	**Mean Difference (95% CI)**	***p*** **-Value**
**ANB, °**	1.20 (1.01)	0.54 (0.50)	0.66 (0.08 to 1.25)	0.03
**SNA, °**	0.90 (0.95)	0.70 (0.59)	0.20 (−0.40 to 0.80)	0.50
**SNB, °**	0.66 (0.49)	0.81 (0.78)	−0.15 (−0.62 to 0.33)	0.54
**Ar-Go-Me, °**	1.09 (1.32)	1.43 (0.98)	−0.34 (−1.19 to 0.50)	0.41
**S-Ar-Go, °**	0.83 (0.97)	0.49 (0.44)	0.34 (−0.22 to 0.89)	0.22

## Data Availability

Not applicable.
